# Study protocol for the DEFENDD trial: an RCT on the Dynamic Locking Blade Plate (DLBP) versus the Dynamic Hip Screw (DHS) for displaced femoral neck fractures in patients 65 years and younger

**DOI:** 10.1186/s12891-020-3131-x

**Published:** 2020-03-03

**Authors:** J. H. Kalsbeek, W. H. Roerdink, P. Krijnen, M. E. van den Akker-van Marle, I. B. Schipper

**Affiliations:** 1 Department of Surgery, Deventer Hospital, Nico Bolkesteinlaan 75, 7416 SE Deventer, the Netherlands; 2 Department of Trauma Surgery, Leiden University Medical Center, Albinusdreef 2, 2333 ZA Leiden, the Netherlands; 3 Department of Biomedical Data Sciences, Leiden University Medical Center, Albinusdreef 2, 2333 ZA Leiden, the Netherlands

**Keywords:** Hip fractures, Dynamic locking blade plate, Dynamic hip screw, Femoral neck fractures, Internal fixation, Gannet, Displaced, DHS, DLBP

## Abstract

**Background:**

The Dynamic Locking Blade Plate (DLBP) was recently introduced for fixation of displaced femoral neck fractures (FNF) and has been well received. Although the results of this implant in young patients are promising, the DLBP has not yet been compared to a standard device such as the Dynamic Hip Screw (DHS). The aim of this study is to compare the clinical outcome and costs of displaced FNF treated with internal fixation by means of either the DLBP or the DHS in patients up to 65 years of age. We hypothesize that the DLBP is superior compared to the DHS in terms of revision surgery rate, union rate, incidence of avascular necrosis and implant related failure.

**Methods:**

The DEFENDD (DisplacEd Femoral Neck fractures Dlbp versus Dhs) trial is a multicentre randomized controlled trial that will include 266 patients of 18–65 years with a displaced FNF. Patients will be randomized to receive either a DLBP or a DHS with a 1:1 allocation using a random block size, stratified for centre. Clinical follow up will last 1 year and questionnaires will be obtained up to 2 years. The main outcome parameter is the incidence of revision surgery within 1 year, due to either non-union, avascular necrosis (AVN) or cut out of the implant. Secondary study parameters are the incidence of avascular necrosis, non-union, (implant related) complications, functional outcome, elective removal of the implant and health-related quality of life and costs.

**Discussion:**

The outcome of the DEFENDD trial will provide high-level evidence of which implant is favourable for the treatment of femoral neck fractures in young patients (≤65 years).

**Trial registration:**

Netherlands Trial Register, NL7300 Registration date 25-09-2018.

## Background

In 1990 an estimated 1.66 million patients sustained a hip fracture worldwide. This number has increased over time and is estimated to be around 6 million in 2050 worldwide [1]. Despite these numbers the optimal treatment of hip fractures is still under debate and subsequently evolving. This especially applies to the treatment of displaced femoral neck fractures (FNFs), which differs considerably worldwide. A general consensus is that young patients (up to 65 years of age) should be treated with fracture reduction and internal fixation [2, 3]. Patients above 75 years of age are in majority treated with arthroplasty. The treatment of FNFs in young elderly (between 65 and 75 years old) is still under debate and is therefore referred to as the ‘unsolved fracture’ [4–6].

**Fig. 1:**
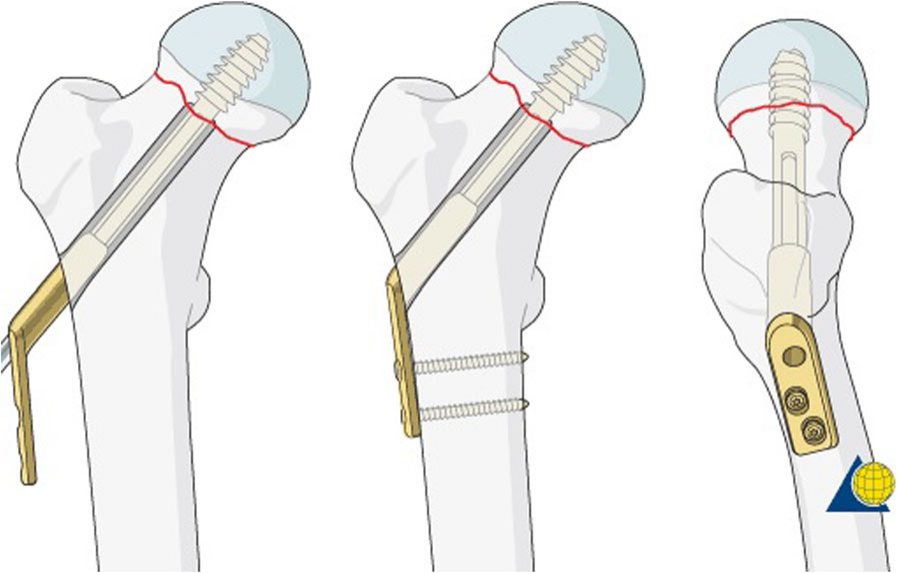
The Dynamic Hip Screw [7]. Copyright by AO Foundation, Switzerland

**Fig. 2:**
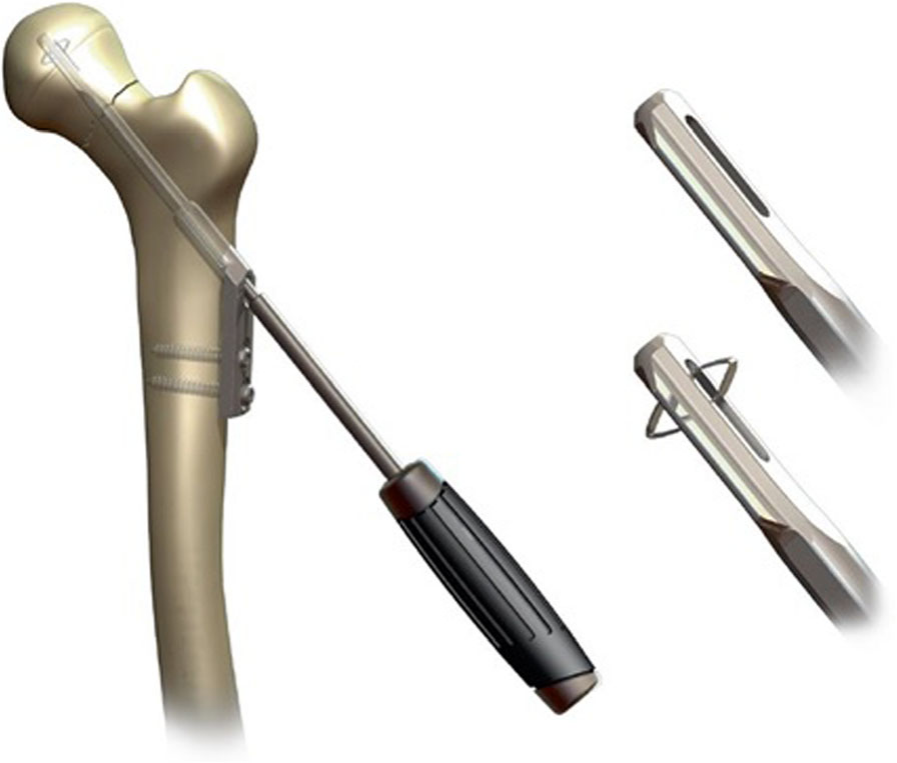
The Dynamic Locking Blade Plate with impaction anchors. Permission was given bij Gannet B.V. Hengelo for using this figure

Nowadays, the most commonly used implants for fixation of FNFs are multiple cannulated parallel screws and the Dynamic Hip Screw (DHS) (Fig. 1). The DHS has a small advantage over multiple parallel screws in displaced FNFs, in that it is known to have a lower reoperation rate [8]. Despite the frequent use of both these implants the failure rate of displaced FNFs is still high, with a non-union rate of 30–33% and an incidence of avascular necrosis (AVN) of 10–16% [3, 9–11]. The reoperation rate, as a result of non-union and AVN is between the 18–48% [3, 11, 12]. The Dynamic Locking Blade Plate (DLBP) (Fig. 2), otherwise called ‘The Gannet’, is specifically designed for the surgical fixation of intracapsular hip fractures though small metal ‘wings’. The characteristics of the DLBP are its low implant volume, rotational stability, angular stability and its simple instrumentation and surgical technique. In a prospective multicenter cohort study in the Netherlands 172 patients with an undisplaced FNF were treated with the DLBP. The results of this study showed a failure rate of 4% [13]. Another recent prospective cohort study of 106 patients of 60 years and younger with displaced FNF demonstrated a DLBP related failure rate of 13.2% [14]. However, randomized controlled trials are needed to provide high-level evidence to determine the value of DLBP.

The aim of the current study is to test if the favorable results with the DLBP persist in a multicenter randomized controlled trial for patients aged 65 years or younger with initially displaced FNFs. We hypothesize that the DLBP is superior compared to the DHS in terms of revision surgery rate, union rate, incidence of avascular necrosis and implant related failure. Also, the cost-effectiveness of the DLBP versus DHS will be assessed.

## Method/design

### Primary and secondary objectives

The primary objective is to test if the incidence of revision surgery (primary endpoint) is lower in patients ≤65 years with an initially displaced FNF treated with the DLBP in comparison to treatment with DHS. Secondary objectives are to determine the incidence of AVN, non-union, implant related complications, non-implant related complications and elective removal after fixation with the DLBP or DHS. Also, we compare operating time, functional outcome and cost-effectiveness of DLBP and the DHS.

### Study design

The DEFENDD trial (DisplacEd Femoral Neck fractures Dlbp versus Dhs) is a multicenter unblinded randomized controlled trial with a superiority design comparing two implants. One group will be treated with the DLBP. The other group will be treated with DHS (control group). The study will be performed in six trauma centers in the Netherlands. Data will be registered in Castor EDC, an online data capture program.

### Eligibility criteria

All consecutive patients between 18 and 65 years with a displaced FNF, Garden type III or IV according to the Garden classification, admitted to the participating hospitals are eligible for the study [15].

Exclusion criteria are:

- Pathological fracture.
- Ipsilateral or contralateral fracture(s) of the lower extremity.
- Injury Severity Score (ISS) of ≥16.
- Local infection or inflammation at time of operation.
- Symptomatic arthritis, diagnosed by a rheumatologist.
- Symptomatic osteoarthritis or radiographic osteoarthritis grade III or IV [16].
- Previous surgery of the ipsilateral hip.
- Open fracture.
- Morbid obesity (BMI ≥ 35).
- Patients who were wheelchair-bound in their pre-injury situation.
- Patients who were, at the time of trauma, admitted to a nursing home.
- Patients who are not mentally competent

### Randomization

After obtaining written informed consent patients are randomized with a 1:1 allocation using a random block size, stratified for centre. Variable block sizes will be determined by the estimated inclusion number of each centre. An online randomization module is used for treatment allocation.

### Sample size calculation

The failure rate or revision rate of the DHS in patients ≤65 years with displaced FNFs described in today’s literature is 32–44% [8, 17]. The failure rate of the DLBP in patients of 60 years and younger with a displaced FNF in a previous cohort study was 13.2% [14]. Analysis of our data showed a failure rate of 15% for the DLBP in patients of 65 years and younger (non-published data). For the determination of the sample size we assumed a 30% failure rate for the DHS and 15% failure rate for the DLBP. For a power of 80% we need 121 patients per group to prove the superiority of the DLBP regarding the primary outcome (need for revision surgery) with alpha of 5% in a two-sided test. Taking into account that up to 10% of the patients may be lost to follow up, 266 patients need to be included for adequate statistical power, i.e. 133 patients per group.

### Study interventions

This trial will be performed in six trauma centres in the Netherlands. In three of the centres the DLBP will be introduced before starting this trial. In the other three participating centres the DLBP is already used. A learning curve is taken into account. The first three DLBP’s in each participating centre will be implanted under supervision of an instructor from the manufacturer or an experienced surgeon who has implanted three or more DLBP’s. The first DLBP’s of a surgeon can be included, provided that they are implanted under supervision.

### Dynamic locking blade plate

The Dynamic Locking Blade Plate consists of a 2-hole standard 135° side-plate combined with a low-volume cannulated dynamic locking blade (Fig. 2). The side plate provides angular stability combined with dynamic axial compression of the fracture. Two side wings at the tip of the blade provide rotational stable fixation of the locking blade in the femoral head combined with a high weight-bearing surface. The expandable impaction anchors lock the blade in the femoral head and prevent perforation and backing out of the implant and further augment the rotational stability. The DLBP is now marketed as the Gannet [13].

### Dynamic hip screw

The control group will be treated with the Dynamic Hip Screw, a stainless steel lag screw in the femoral neck and head that is fixated to the femur shaft with a compression plate using two-four 4,5 mm cortical screws (Fig. 1). The DHS is used globally and is provided by a wide range of commercial producers in various sizes. It can be implanted with or without an additional cannulated antirotational screw. The type of DHS used in the control group is at the discretion of the surgeon. The trauma and orthopaedic surgeons in participating trauma centres have a wide experience with internal fixation of FNF by means of DHS.

### Direct post-operative care

Both groups receive standard care including direct mobilization after surgery. Mobilization therapy will be given by a physiotherapist according to the hospital protocol for hip fracture after care. All patients receive low-molecular-weight heparin anticoagulation therapy during their stay in the hospital.

### Study procedures

A time schedule of procedures and measurements is presented in Table 1. The selection of eligible patients will take place in the emergency department (ED). According to standard care, X-ray examinations of the pelvis and hip are made on admission and assessed by the radiologist and trauma surgeon. Eligible patients will receive oral and written information about the study from the physician in the ED. The patients have at least 6 h to consider participation in the study and will be given the opportunity to ask questions about the study. Written informed consent will be obtained by the surgical resident or the surgeon after admission to the ward. Randomization will be done by the treating surgeon. After inclusion the patient will be allocated to one of the two study groups (DLBP or DHS) using an online randomization program. The baseline parameters will be registered by a nurse upon arrival on the surgical ward before surgery. The perioperative care will be the same for all included patients.

**Table 1:** Time schedule for study procedures and measurements

	Emergency department	Admission	Post-op visit (≤5 days)	6 weeks follow-up	3 months follow-up	6 months follow-up	12 months follow-up	24 months follow-up^a^
Informed consent		x						
Baseline characteristics		x						
Radiography	x		x	x	x		x	
Questionnaires		x		x	x	x	x	x
Complications registration		x	x	x	x		x	x

^a^ Contact by telephone

Surgery will be performed by an (orthopaedic) trauma surgeon or by an (orthopaedic) trauma resident under the direct supervision of an (orthopaedic) trauma surgeon. The aim is to operate within 24–36 h based on the Dutch guidelines for treatment of FNFs [18]. After surgery, details about the surgery will be documented.

After discharge patients are scheduled for outpatient visits after 6 weeks, 3 and 12 months. Conventional radiographs will be taken and assessed during these visits (Table 1). The patients need to fill out a questionnaire before follow up visits and 6 months after discharge. Also, the patient will be contacted by telephone 24 months after enrolment in the study for additional questionnaires about mobility and complication registration.

### Study parameters

#### Primary outcome parameter

The primary outcome is the incidence of revision surgery after fixation of an initially displaced FNF treated with DLBP or DHS due to non-union, AVN or cut out of the implant. This will be monitored during 1 year of follow up after surgery.

#### Secondary outcome parameters

*Incidence of avascular necrosis*: AVN is defined as hip pain in combination with radiographical signs for AVN as described by Steinberg [19]. According to the Steinberg classification AVN is present from stage 2 and upward. AVN will be assessed by the treating surgeon. As is customary in the Netherlands, all radiographs are also assessed by a radiologist.

*Incidence of non-union*: there is no consensus in the literature regarding to the definition of (non-)union [20]. Our definition of non-union is based on the Radiographic Union Score for Hip (RUSH) [21]. Non-union is a visible fracture line on the radiograph, absence of cortical bridging or bridging trabeculae over the fracture site in combination with persisting pain in the hip and the inability to bear weight at least 9 months post-operative or sooner if revision surgery was performed because it was no longer expected that fracture healing would occur. Non-union will be assessed by the treating surgeon.

*Incidence of implant related complications:* an implant related complication is defined as breakage or cut-out of the plate or screws, inadequate expansion/malfunction of the anchors or any malfunction of the implant which may or may not lead to revision surgery. Implant related failure will be monitored during 1 year of follow up.

*Post-operative complications*: post-operative complication is defined as any unanticipated event other than the above mentioned, for which operative treatment or medical treatment is required, e.g. wound infection, bleeding or pneumonia. Every complication occurring during the hospital stay of the patient will be recorded.

*Rate of elective implant removal after union*: Elective implant removal after union will be recorded during 1 year of follow up after surgery. Reasons for elective removal will be described.

*Functional outcome:* patient-reported post-surgical function will be scored using the validated Dutch version of the International Hip Outcome Tool (iHOT-12NL) [22]. The iHOT-12NL is a patient-reported questionnaire that measures health-related quality of life and physical function in younger, active patients with hip disorders. Scores on the iHOT-12NL range between 0 and 100 (worst - best possible function). This questionnaire will be filled out by the patient during admission and at 6 weeks, 3, 6 and 12 months follow up.

*Operation time*: the operation time is recorded in the surgical report.

*Baseline parameters:* Additional parameters that will be recorded are: sex, date of birth, general health score (using the ASA classification), fracture type and side, trauma surgeon or orthopaedic trauma surgeon, type of anaesthesia, Body Mass Index. These parameters will be assessed during admission as a baseline measure.

*Costs*: Costs will be assessed from a societal perspective. Cost of (revision) surgery will be calculated using a bottom-up approach. Using a questionnaire the patients will report other health care use such as physiotherapy, rehabilitation care or nursing home care, visits to the general practitioners and medical specialists and medication, and non-medical care (domestic help and absenteeism). This questionnaire will be filled out by the patient at 6 weeks, 3, 6 and 12 months follow up. Health care use will be valued using Dutch reference prices [23].

*Health related quality of life*: the EuroQol (EQ-5D-5 L) questionnaire measures five dimensions (mobility, self-care, daily activities, pain/discomfort, anxiety/depression), on a five-point scale (no, some, moderate, much or extreme problems). For each health state described by the patients, a utility score can be calculated that reflects society’s valuation of that health state [24]. In addition, patients rate their overall health-related quality of life on a Visual Analogue scale (VAS). This questionnaire will be filled out by the patient during admission and at 6 weeks, 3, 6 and 12 months follow up. The utility scores obtained by the descriptive system and the VAS will be used in the cost-effectiveness analysis.

### Statistical analysis

Statistical analysis will be performed using SPSS (IBM Corp., Armonk, NY, USA). Primary analysis will be done according to the intention-to-treat principle. If patients are not treated according their allocated treatment a per-protocol analysis will be conducted to confirm the intention-to-treat analysis. Baseline characteristics of the treatment groups will be presented as mean with SD or as median and range for continuous variables and as number and percentage for categorical variables.

The primary outcome parameter, the incidence of revision surgery after 1 year, will be compared between the treatment groups using logistic regression analysis, including study centre as a covariate, since some study sites have used the DLBP for several years and whereas in other medical centres the DLBP has only been introduced recently. In literature there is no clear evidence of other covariates that have a strong or moderate association with the primary outcome. The secondary parameters: the incidence of AVN, non-union, implant-related complications, post-operative complications, and elective implant removal after 1 year will be analysed in the same manner as the primary outcome parameter. Operation time will be compared between the treatment groups using the independent samples t-test or the Mann-Whitney test, as appropriate. Functional outcome at the specified follow-up moments will be compared between the treatment groups using an independent samples t-test. In addition, the course of functional recovery over time will be compared using a linear mixed model with time, treatment and baseline characteristics as fixed effects, and patient as random effect. Missing data will be imputed using multiple imputation before testing the differences in the outcome parameters. *P*-values less than 0.05 will be considered statistically significant.

The economic evaluation will compare differences in societal costs, as described in the paragraph ‘Study parameters’, to differences in quality adjusted life years (QALYs). Utilities obtained from the EQ-5D-5L will be used to determine QALYs. The QALYs will be calculated from the area under the curve in a utility-time figure. The duration of the trial will be taken as the time-horizon. Group averages will be statistically compared using non-paired t-test and a net-benefit analysis will be used to compare costs to patient outcome. Results will be presented in a cost-effectiveness acceptability curve.

### Monitoring

Patient data will be handled confidentially and in compliance with the Dutch Personal Data Protection Act. Collected data will be stored in Castor EDC, an electronic data capture program. Stored data will be coded, using a unique combination for centre and successive study number. The key to the code will be accessible by the local investigators and the coordinating investigators. Study data will be kept for 15 years and destroyed afterwards. The local investigators will have access to the link between code and personal data of the patients of only his centre. The coordinating and the principal investigator have access to all the data. The co-investigators will have access to the coded data of all patients.

The coordinating investigators will report all adverse events to the accredited Medical Research Ethics Committee (MREC) that approved the protocol. No data safety managing board is installed. The investigator will submit a summary of the progress of the trial to the accredited MREC once a year. Information will be provided on the date of inclusion of the first subject, numbers of subjects included and numbers of subjects that have completed the trial, serious adverse events/ serious adverse reactions, other problems, and amendments. No planned interim analyses will be conducted.

## Discussion

In this paper we present the rationale and design of a randomized controlled trial that compares the clinical outcomes of the DLBP and the DHS. The DHS is a globally accepted osteosynthesis and it has been for decades. Yet the failure rate is high. The DLBP is a new implant that is on the market since 2010. Today’s evidence for this implant is not as widespread as for the DHS, but the results from (non randomized) earlier studies are promising [13, 14]. The outcome of the DEFENDD trial will provide high-level evidence of which implant is favorable for the treatment of femoral neck fractures in young patients (≤65 years). The results of this trial will be published in peer-reviewed international journal.

## Data Availability

The datasets generated and/or analysed during the current study are not publicly available but are available from the corresponding author on reasonable request.

## Abbreviations

AVN: Avascular necrosis
BMI: Body mass index
DHS: Dynamic Hip Screw
DLBP: Dynamic Locking Blade Plate
ED: Emergency department
FNF: Femoral Neck Fractures
GCP: Good Clinical Practice
iHOT: International Hip Outcome Tool
MREC: Medical Research Ethics Committee
QALY: Quality Adjusted Life Years
RCT: Randomized Controlled Trial
WMO: Medical Research Involving Human Subjects Act (in Dutch: Wet Medisch-wetenschappelijk Onderzoek met Mensen)
